# Involvement of Nuclear Factor-κB in Inflammation and Neuronal Plasticity Associated with Post-Traumatic Stress Disorder

**DOI:** 10.3390/cells11132034

**Published:** 2022-06-27

**Authors:** Sudhiranjan Gupta, Rakeshwar S. Guleria

**Affiliations:** Biomarkers & Genetics Core, VISN 17 Center of Excellence for Research on Returning War Veterans, 4800 Memorial Drive (151C), Waco, TX 76711, USA; rakeshwar.guleria@va.gov

**Keywords:** PTSD, NF-κB, inflammation, neuronal plasticity

## Abstract

Post-traumatic stress disorder (PTSD) is a debilitating psychiatric condition which develops either due to stress or witnessing a traumatic situation. PTSD is characterized by acute and chronic stress response exhibit anxiety, fear, and an increased inflammatory etiology. Inflammation contributes a critical role in several parts of the brain that control fear and flashback cognatic function. It is known that impairment of the neurological circuit leads to the development of PTSD. Evidence has suggested that dysregulation of the sympathetic nervous system and hypothalamic-pituitary adrenal (HPA) axis and inflammatory responsiveness are pivotal and a greater risk in PTSD. NF-κB, a master regulator for inflammation, has been showed to modulate memory reconsolidation and synaptic plasticity; however, NF-κB’s association with PTSD remain elusive. In this review, we provide relevant findings regarding NF-κB activity in various components of brain and describe a potential mechanism linking PTSD using preclinical and clinical models. We envisage NF-κB signaling as a crucial mediator for inflammation, cognitive function, memory restoration and behavioral actions of stress and suggest that it could be used for therapeutic intervention in PTSD.

## 1. Introduction

Post-traumatic stress disorder (PTSD) is a severe distressing condition that develops in a subset of individuals after a major traumatic event and is associated with high morbidity and mortality [1,2]. In addition, PTSD is linked with deregulation of the sympathetic nervous system, hypothalamic-pituitary-axis (HPA) [3,4,5,6]. Recent evidence suggests that dysregulation of inflammation significantly contribute to development of PTSD [7,8]. However, the interplay between inflammation and neurocognitive pattern in PTSD is emerging. In the settings of therapeutic intervention, a deeper understanding of inflammatory pathology would be of interest for behavioral intervention.

In this review, we first describe pathophysiology of PTSD, then summarize evidence of dysregulation in the inflammatory circuit system in PTSD. We further describe the potential role of NF-κB, a master regulator of inflammation linking PTSD with the dysregulated inflammatory system by examining relevant findings from basic and clinical studies.

## 2. PTSD and the Pathophysiology

PTSD is a debilitating condition and one of the most pervasive psychiatric conditions resulted from traumatic stress. The term PTSD was first used in the third edition of the Diagnostic and Statistical Manual of Mental Disorders and was classified as an anxiety disorder [9]. In a subsequent revision in 1987, the diagnostic criteria were modified and emphasize the avoidance phenomena [10]. The inherent characteristics of PTSD are the alteration of mood, avoidance, increased arousal, hypervigilance, sleep disturbance, cognition deficit, flashbacks, reexperiencing traumatic events and persistence of intense fearful reactions [11]. PTSD is also a serious burden in deployed combat Veterans [12]. PTSD is frequently diagnosed in service members returning from war zones [13,14,15]. Typically, four essential features are examined during diagnosis and, they included traumatic events that caused injury, revisiting the angst periods with flashbacks, avoidance of past frightful memories and increased arousal. A significant increase of PTSD is observed in socially challenged people, young people, and first responders to trauma [16] and is associated with mood, anxiety and substance related issues [17]. Therefore, it is imperative to understand the alteration of brain physiology and the possible mechanism that caused the traumatic stress for therapeutic intervention.

The pathophysiology of PTSD necessitates the changes in neurotransmitter release and neurohormonal function in the brain. It is noted that an individual with PTSD showed a low level of glucocorticoid or cortisol [18] and high level of corticotrophin releasing hormone (CRH), a critical stress hormone [19]. The high level of CRH is observed in the cerebrospinal fluid indicated in PTSD patients indicated their association with the severity of the disorder [20,21]. In response to stress, hypothalamus releases CRH from paraventricular nucleus which activates norepinephrine neuro-modulatory system and triggers the behavioral “fight or flight” response from the SNS [22,23]. CRH acts on the pituitary gland, which in response secretes adrenocorticotropic hormone (ACTH) into the bloodstream. Once ACTH reaches the adrenal glands, it triggers the release of cortisol (in humans) or corticosterone (in rodents), which acts as anti-inflammatory hormones, and coordinates the physiological behavioral response to stress. Interestingly, single nucleotide polymorphism (SNP) in CRF1 receptor gene is reported to modulate stress susceptibility or resilience in severe depression [24,25]. Furthermore, SNP in the CRF1 receptor gene is reported in PTSD patients suggested that CRF signaling contributed to depression-related cognitive dysfunction pediatric PTSD subjects [26].

Essentially, two central physiological pathways are involved in PTSD; the hypothalamic pituitary adrenal (HPA) and sympathetic nervous system (SNS). The HPA-axis is a nodal point of major regulatory pathways controlling physiological and biochemical responses to stress. Alterations in glucocorticoid receptors (GRs) in HPA axis are implicated in the pathogenesis of PTSD [27]. Physiological stimuli or stress trigger HPA-axis leading to the secretion of glucocorticoid (GC) from adrenal cortex and mediating a negative feedback circuit during stressful situation [28]. Studies showed varied GR numbers, GR promoter methylation in PTSD conditions [29,30,31,32], however, an increased sensitivity of GR and inflammation are observed in PTSD cohorts [33,34].

The focal point of the central nervous system involved in fear is the amygdala. The almond-shaped mass in the cerebral hemisphere of brain structure primarily governs our ability to experience fear, emotions and extinguish memory of fearful stimuli [35,36]. The role of the amygdala in the pathophysiology of PTSD is still incompletely understood, however, recent functional magnetic resonance imaging (fMRI) technique has shown the ability to identify increased amygdala activity in PTSD following exposure to traumatic stress [37]. Patients with PTSD compared to non-PTSD subjects showed greater amygdala activation during exposure to an aversive stimulus and during extinction training [38,39]. Moreover, amygdala volume, a morphological feature in the structural plasticity of amygdala, is considered as critical factor in PTSD. The research conducted by Kuo et al. [40] using veterans with PTSD demonstrated a decreased amygdala volume in PTSD compared to the control group. Similar studies further confirmed the finding and claimed a relationship between smaller amygdala volume and stronger fear or stress response [41]. Recent studies showed that inflammatory response may be a contributor to enhanced amygdala activity. Neuroimaging studies demonstrated that increased amygdala activity to stress is associated with IL-6 secretion [42].

## 3. Neuronal Plasticity in PTSD

Neuronal plasticity may be defined as the ability to adapt nerve cells in the brain through changes in the growth, remapping or reorganizing of the neighborhood regions. It is also suggested that molecular reorganization of synapse may occur due to structural alteration of neuron in interneural communication process [43,44]. The brain areas that are involved in PTSD are prefrontal cortex, amygdala and hippocampus; tangled with long-term changes in the neurobiology of the brain. The neurohormonal machinery primarily act on brain areas to control PTSD symptoms like GR and norepinephrine. Therefore, a dysfunction in synaptic or neuronal plasticity with varied form is pivotal in PTSD symptoms. Below, we will discuss brain areas that are affected by plasticity.

### 3.1. Prefrontal Cortex and PTSD

The PFC participates in numerous cognitive functions including memory, processing, decision making, and behavior [45]. PFC is a heterogeneous structure and contains four functional parts based on dorsal/ventral and lateral/medial locations constituting dorsomedial prefrontal cortex (dmPFC), ventromedial prefrontal cortex (vmPFC), dorsolateral prefrontal cortex (dlPFC) and ventrolateral prefrontal cortex (vlPFC) regions. Both dmPFC and vmPFC regions are active in normal memory and fear processing, but, in PTSD cases, it is noted smaller activation particularly in war veterans [46]. Dysregulation of dlPFC and connection distribution in the cortical region resulted in hypoactivation in PTSD and considered as critical contributor in PTSD [47]. The repetitive transcranial magnetic stimulation (rTMS), a non-invasive stimulation using repetitive magnetic pulses in the brain tissue, used for various psychiatric diseases including PTSD, showed significant reduction of PTSD symptoms by targeting dlPFC [47]. Another veteran study reported that severity of PTSD is inversely correlated with anterior cingulate cortex volume [48]. However, the cause for hypoactivity of vmPFC is due to PTSD or a secondary effect is undetermined. Studies have also shown that miscommunication or disruption of vmPFC-amygdala was pivotal in the pathogenesis of PTSD and symptoms [49]. Also, Interestingly, studies demonstrated a reduction in structural integrity of white matter tracts in PTSD patients using diffusion tracer imaging [50]. The uncinate fasciculus white matter tracts, a long-range fiber tract that connects vmPFC to multiple subcortical, limbic regions including amygdala, has been proposed to play a role in language recognition. These facts together supports that dlPFC dysregulation in PTSD and targeting dlPFC structure and function possibly helps to ease PTSD symptoms. Therefore, structural and functional alteration in PFC in PTSD is critical and further research is warranted.

### 3.2. Amygdala and PTSD

The amygdala is an almond-shaped mass situated in the cerebral hemisphere controlling emotion. It is one of the key brain components necessary in the pathophysiology of PTSD. It is subdivided into 13 or more subnuclei; of which basolateral amygdala (BLA), central amygdala (CeA) and medial amygdala (MeA) were well documented [51]. The BLA contains excitatory neurons showing cortical-like profile whereas the CeA and MeA show striatal-like composition and are inhibitory neurons [50]. A greater engagement of amygdala activity is seen in PTSD patients [52,53,54]. An increased amygdala activity was conformed in fMRI study [55]. Similar activity was reported in Vietnam war veterans [46]; however, no activation was reported in conditions with PTSD [56]. Furthermore, the investigation of PTSD and amygdala volume are debatable; however, a trend showed smaller volume [57,58,59]. It is still unclear whether a smaller volume of amygdala is the outcome of PTSD or represented as a preexisting situation. It is therefore imperative to understand the role of amygdala activation or volumetry in the pathophysiology of PTSD, which is currently less understood.

### 3.3. Hippocampus

Hippocampus, a S-shaped structure and an integral part of limbic system plays a vital role in memory regulation. It is embedded in the temporal lobe of each cerebral cortex. It is a brain memory navigation module that is divided into three parts or cornu ammonis (CA) namely CA1, CA2 and CA3, respectively, constituting a long-term memory system [60]. Similar to amygdala, individuals with PTSD exhibit greater hippocampal activation during emotion processing [52]. A smaller hippocampal volume has been considered a risk factor to develop PTSD in women [61]. The integrity of hippocampus and its morphometric correlates are vital in degree or severity of PTSD [62,63]. In addition to CA, the hippocampus is divided two other subfields, the dentate gyrus (DG) and subiculum [64]. In the hippocampus, DG plays a critical role in learning, memory, pattern separation meaning ability to lessen the resemblance between two similar memories, contextual and spatial information [65]. Hippocampal changes specifically causing a reduction in DG volume was reported in a PTSD individual [66,67]. Furthermore, an association between smaller DG and major mood disorder was reported in postmortem MDD subjects, indicating its pivotal role in PTSD [68]. Together, this underscored that DG plays a role in maintaining PTSD symptoms, and future studies are required for deeper understanding of whether low volume of deficit of DG is the cause of PTSD.

Together, the above components of the brain underscore the importance of their function in the pathophysiology of PTSD. The morphological correlate, the size, or the volume appear to be critical in hyperarousal or negative behavioral events in PTSD.

## 4. NF-κB and PTSD

### 4.1. Inflammation and PTSD

The concept of inflammation in mental disorder or major depressive disorder appears to be unknown. However, there might be a link when an observation was published, dating back as an offshoot of interferon α treatment in chronic hepatitis C patients [69]. The study showed that 17% of patients developed psychiatric side effects after a year-long treatment with recombinant interferon α [69]. Subsequent studies have identified an association between immune deregulation and inflammatory response in several psychiatric disorders, including PTSD [70].

Inflammation is a predominant cellular process derived from cellular injury and it diseases with a goal to protect the injured site by releasing several inflammatory cells like neutrophils, monocytes or lymphocytes. These cells release several chemical mediators including enzymes and cytokines to alleviate the response [71]. Emerging evidence suggests that inflammatory condition can be activated by chronic psychological stress or mental illnesses, including PTSD, and may exert destructive effects on the brain. Importantly, inflammation is suggested to be a pivotal event of PTSD in combat veterans [72]. Findings from blood biomarkers along with genetic variants of several genes indicated that inflammation appears to be a key process in the pathology of PTSD. The association between PTSD and inflammation were observed primarily in blood inflammatory molecules including a wide spectrum of cytokines, and c-reactive protein (CRP), etc. Over the past several years, several studies have shown that an individual with PTSD elicits an enhanced level of blood cytokines such as IL-1β, IL-6, TNFα, etc. [73,74,75,76,77]. Interestingly, IL-6 appeared to be the most significant cytokine in PTSD individuals [7,78]. In addition to inflammatory blood biomarker identification, inflammatory genetic association or gene expression in PTSD are less studied. Bruenig et al. showed a polymorphism in TNFα gene promoter [TNFA -308 (rs1800629)] in PTSD in Vietnam war veterans [77]; however, the link between PTSD and TNFα polymorphism is not established. Another genome-wide association study of PTSD showed a link between inflammation and PTSD and single-nucleotide polymorphism (SNP) in the retinoid-related orphan receptor α (*RORA*) gene, rs8042149 [79]. Therefore, variants within inflammatory genes that encode cytokines, such as *TNFα*, *IL-6* and *IL-1β*, are warranted for further investigation in PTSD.

Another well-studied inflammatory marker is CRP. Studies have shown a positive correlation between CRP and PTSD where individuals with PTSD showed increased CRP levels compared to healthy controls [73,78,80,81]. However, mixed results were observed in different PTSD cohorts. No significant difference was observed between control and PTSD individual in a meta analyzed by Passos et al. [78,82]. Recent studies have reported that the severity of CRP and PTSD symptoms may not be considered as a specific biomarker for PTSD [83,84]. The etiology and pathophysiology of PTSD is complex and in this setting one possible reason for variability may lie in the genetic variant or SNPs. Ref. [85] have identified CRP SNP *rs1130864* to be associated with PTSD in a large urban sample with PTSD symptoms. However, they did not examine *CRP* SNPs and the severity of PTSD and CRP levels. The findings were further analyzed by Miller et al. who suggested that “*CRP* SNPs moderated the association between PTSD and CRP levels” [86].

The mechanism of inflammation and PTSD is unclear and complex [87]. One of the common mechanisms suggested altered HPA-axis and autonomous nervous system (ANS) [6,88]. The hypoactive HPA-axis and hyperactive of sympathetic nervous system (SNS) appear to a plausible cause for PTSD. Evidence indicated that dysregulation of the HPA-axis resulted in low cortisol level in the blood eventually developing an inflammatory state [89]. It is noted that cortisol is glucocorticoid and is an anti-inflammatory and immunosuppressive agent [90]. Glucocorticoid further autoregulates its level via negative feedback mechanism by binding to glucocorticoid receptors in the hypothalamus and pituitary. Furthermore, stress triggered the release of corticotrophin hormone (CRH) in the hypothalamus that stimulates SNS to produce catecholamines including norepinephrine, a prime cause for hyperarousal in PTSD [90]. Therefore, deregulation of the HPA-axis can influence chronic inflammation (low-grade) in PTSD.

Another mechanism postulated that the stress associated proinflammatory condition created a “sterile inflammation”, the inflammation in the absence of pathogenic response, triggered by the activation of pattern recognition receptors (PRR) which bind to damage (or danger)-associated molecular patterns (DAMP) [91,92]. DAMPs are endogenous molecules which increase in response to psychological stress and include a variety of different ligands, like heat-shock proteins, S100 proteins, high mobility group box 1, uric acids, and adenosine triphosphate, which have been shown to promote PTSD like condition in a stress model [7,92].

Overall, previous studies have suggested that there is a strong correlation between inflammation and PTSD. It is imperative to understand how peripheral inflammation influences neurocognitive function, including attention, processing and executive function in PTSD. Evidence has suggested that peripheral or central inflammation negatively affected cognitive response [93]; however, studies are limited in the setting of deeper understanding of inflammation and neurocognitive deficit in PTSD. Thus, we propose further research in the line of inflammatory response, clinical assessment and neurobiology of PTSD.

### 4.2. NF-κB and PTSD

The nuclear factor κB (NF-κB) is a ubiquitous transcription factor that regulates a wide array of cellular and molecular functions in diverse diseases. Since its discovery in 1986 by Sen and Baltimore [94] in immune cells (B-cells), it showed promise in many therapeutic interventions. In addition to its critical role in immune modulation and inflammation, NF-κB gained attention in the nervous system as it showed a pivotal role in neuronal plasticity, memory formation, synaptic processes, neurotransmission, and neuroprotection [95,96,97,98]. The central nervous system (CNS) hosts an array of different type of heterogenous cell population and the interplay between cell types and NF-κB activation is largely unknown. Specifically, NF-κB’s role in PTSD is largely unknown. A recent review by Dresselhaus and Meffert [99] illuminate the contribution of NF-κB in CNS.

The NF-κB family is composed of several members. In mammals, the NF-κB family comprises five members: NF-κB1 (p105/p50), NF-κB2 (p100/p52), RelA (p65), RelB, and c-Rel. NF-κB1 and NF-κB1 are synthesized as large polypeptides, separately, and p50 and p52 subunits are generated after the posttranslational cleavage. Under normal condition, NF-κB (mostly p50 and p65) is sequestered in the cytoplasm with its inhibitory partner, IκB proteins, primarily IκBα and IκBβ [100,101,102]. Upon stimulation, IκB is phosphorylated by the IκB kinase complex, the IKK, ubiquitinated, and consequently degraded by the 26S proteasome. The released NF-κB, translocated into nucleus, binds to a specific DNA element and activates several NF-κB dependent genes [101,103]. A wide range of factors including tumor necrosis factor α, interleukin-1, nerve growth factor, lipopolysaccharides, reactive oxygen species can activate NF-κB and depending on the type of stimulus the posttranslational modification occurs [104].

The role of NF-κB in the nervous system in not unknown. NF-κB modulates several physiological, pathological and development functions in chronic neurological disorders [105,106,107]. However, in a rodent model, it has been reported that NF-κB is activated in ischemia and neurodegenerative diseases [108,109,110]. Several studies have also documented increased levels of NF-κB activation in brain tissues of traumatic brain injury, focal ischemia and seizure [111,112,113]. Furthermore, NF-κB activation was observed in neuronal survival [114], and blockade of NF-κB showed loss of neuroprotection in neuron-specific deletion of NF-κB [115]. However, the role of NF-κB in PTSD remains elusive although few reports showed NF-κB in psychological rodent models. Kassed, C. et al. [116] for the first time reported lesser anxiety-like behavior using p50 null mice. Additionally, psychological rodent models showed that NF-κB played a critical role in anti-neurogenic and behavioral actions and suggested therapeutical targets for depression [117]. Another study using the predator scent stress rat model showed long-term NF-κB activation in the hippocampus and elicited PTSD-like behavioral coming up were reduced using NF-κB inhibitor (PDTC) and high dose of corticosterone [118]. The study is interesting as inhibition of NF-κB restored the behavioral process in a stress model mimicking PTSD. Moreover, increased NF-κB activation was observed in major depression patients with increased early life stress indicated a link between major depression, early life stress, and inflammation [119]. Future studies are warranted for the treatment of stress related clinical disorders.

In a clinical setting, there is no direct measurement of NF-κB activity in patients with PTSD. However, one study investigated NF-κB signaling using monocytes from PTSD individuals [120]. The authors examined specific transcription binding motifs in the promoter regions of differentially expressed genes in monocytes from PTSD patients, compared with healthy controls. The authors showed upregulation of NF-κB target genes in male and female patients with PTSD. In addition, authors observed downregulation of GR target genes in these patients. The study indicated that altered monocytes gene expression may influence inflammatory pathways in PTSD patients [120] as NF-κB is a master regulator for inflammation.

## 5. A predictive Scenario of NF-κB Activation in PTSD

It is reported that proinflammatory molecules are synthesized and released from several cell types in CNS including astrocytes, microglia and neurons along with immune cells like macrophages [121]. Stress triggers the synthesis of CRH in the paraventricular nucleus of the hypothalamus and stimulates further to SNS to produce catecholamines, including norepinephrine, which leads to the induction of a castade of proinflammatory cytokines, such as IL-1 and IL-6, via an NF-κB-dependent manner [122]. On the other side, GR, a 777 amino-acids nuclear receptor, modulates the transcription of many genes in the HPA-axis [123,124,125]. The GR binds to GC at its C-terminal ligand binding site, dimerizes and translocates into the nucleus, binds the GC responsive element (GRE) with the help of co-activators like GRIP1, and activates transcription [126,127]. Glucocorticoids are anti-inflammatory molecules and are known to bind to other transcription factors like NF-κB-p65 [128,129,130]. The mechanism of GR and NF-κB interaction has been extensively studied, and GR is shown to interact with NF-κB protein, (p65) via protein-protein interactions and impaired NF-κB transactivation [129,130,131]. Both GR and NF-κB activity is seen in several parts of brain regions during stress. During inflammation, it is observed that both GR and NF-κB are involved in glial cell activation, possibly due to presence of immune receptors that facilitate the synaptic plasticity [132]. Synaptic plasticity is altered during chronic stress [4,133,134]. During stress, proinflammatory and anti-inflammatory molecules are released by microglia and may alter synaptic plasticity [135]. The inflammatory condition may lead to compromised glial function by disrupting glutamate homeostasis [136]. It is also noted that both GR and NF-κB are present in neurons and thought to play a role in brain development and synaptic signaling [97,98,137,138]. It is reported that, in a chronic stress population, a reduced GR and increased NF-κB activity is observed [139,140]. The activation of NF-κB is linked with long-term synaptic plasticity, and long-term memory, and was found to be activated in long-term potentiation in rodent study [141,142]. Treating the mouse brain with NF-κB decoy prevented the long-term depression and reduced LTP (full form of LTP) [143]. Similarly, in p50, knockout model late-LTP is impaired and it is suggested that NF-κB-p50 is required for long-term spatial memory in the hippocampus [144]. Similarly, GCs are anti-inflammatory and immunosuppressive in nature and have been found to induce *IκBα* expression and blocked nuclear translocation of NF-κB in chronic rat stress model [145]. Therefore, the imbalance of GR and NF-κB interplay may be pivotal in a psychiatric related disease like PTSD and are suggested for the development of targeted strategy to combat inflammation.

Fear memory is one of the pivotal phases in acute and chronic PTSD, and research has been trying to reconsolidate fear memory to alleviate PTSD symptoms. In a rodent model it has been shown that NF-κB is critical in synaptic plasticity, animal behavior and long-term memory formation [146,147], and inhibition of NF-κB dampens the reconsolidation of memory [148]. Specifically, the subunits of p50 and p65 were involved in memory formation and synaptic activity [149,150]. Amygdala, a central region for fear conditioning in PTSD has shown that NF-κB activity is required in the basolateral amygdala for memory reconsolidation and inhibition of NF-κB disrupted the process [151]. The observation indicated that NF-κB might be a potential pharmacotherapy target for PTSD.

Microglia played a pivotal role in immune, synaptic plasticity and cognitive function by surveying neuronal environment, but various neurological disorders, including PTSD, disrupt the microglial function [152,153,154,155]. Microglial dysfunction regulates cognitive deficits associated with PTSD. A number of PTSD studies showed increased proinflammatory molecules [78], which were secreted mainly from activated microglia to combat the situation and returned to resting state [152,153]. However, in a chronic state, the microglia changed the morphology and become dysfunctional [153]. In an electric foot-shock PTSD mouse model, microglia are shown to be activated, and an increase in the number of microglia altered morphology, and reduced branches and dendritic spine density in the CA1 region of the hippocampus and Pfc in the Cx3cr1-GFP mice [153]. The changes are the reflection of synaptic dysregulation and memory impairments.

In summary, it may be suggested that, during PTSD, a dysfunctional HPA axis increased CRH to stimulate adrenocorticotropin secretion, leading to an imbalance in cortisol level that alters synaptic plasticity, activating NF-κB signaling and releasing proinflammatory cytokines.

## 6. Conclusions

Our review revealed that inflammation is prevalent in the PTSD population, and different parts of the brain serve as modules to orchestrate the neurological signal affecting the etiology of PTSD and reflecting the cognitive function. NF-κB signaling is critical in long-term memory formation, synaptic plasticity, proinflammatory cytokines surge, and behavioral function in the brain. Blockade of NF-κB mitigated the proinflammatory cytokines implicated in stress and depression, and could provide beneficial actions for the treatment of PTSD. Furthermore, there is convincing evidence supporting the role of NF-κB in synaptic plasticity, and memory reconsolidation in rodent models indicated its critical role in LTP induction and memory retention, particularly in amygdala. Collectively, the current data underscore a plausible role of NF-κB in modulating synaptic niche and coordinating the inflammatory response in acute and chronic stress situations in PTSD. The findings may provide a novel target for pharmacological intervention in an individual with PTSD. However, there are open questions for the future determination of NF-κB’s role in PTSD. First, a limited number of studies have examined NF-κB‘s contribution in human subjects and more PTSD cohorts are required to validate the observation. Second, NF-κB associated gene expression in the setting of cellular stress and inflammation during synaptic plasticity warrants further investigation and it could provide more insight at an organ-specific gene regulated network in the brain. Third, the selective regulation of NF-κB in learning and memory formation needs more in-depth information in the setting of cognitive behavior in PTSD. Fourth, the context of NF-κB signaling as anti-neurogenic and behavioral actions of stress requires further study.

## Data Availability

Not applicable.

## References

[1] Shalev A., Liberzon I., Marmar C. (2017). Post-Traumatic Stress Disorder. N. Engl. J. Med..

[2] Edmondson D.E., Kronish I.M., Shaffer J.A., Falzon L., Burg M.M. (2013). Posttraumatic Stress Disorder and Risk for Coronary Heart Disease: A Meta-analytic Review. Am. Heart J..

[3] Andrews J.A., Neises K.D. (2012). Cells, biomarkers, and post-traumatic stress disorder: Evidence for peripheral involvement in a central disease. J. Neurochem..

[4] He M., Wei J.X., Mao M., Zhao G.Y., Tang J.J., Feng S., Lu X.M., Wang Y.T. (2018). Synaptic Plasticity in PTSD and associated Comorbidities: The Function and Mechanism for Diagnostics and Therapy. Curr. Pharm. Des..

[5] Speer K.E., Semple S., Naumovski N., D’Cunha N.M., McKune A.J. (2019). HPA axis function and diurnal cortisol in post-traumatic stress disorder: A systematic review. Neurobiol. Stress.

[6] Dunlop B.W., Wong A. (2019). The hypothalamic-pituitary-adrenal axis in PTSD: Pathophysiology and treatment interventions. Prog. Neuropsychopharmacol. Biol. Psychiatry.

[7] Hori H., Kim Y. (2019). Inflammation and post-traumatic stress disorder. Psychiatry Clin. Neurosci..

[8] Sumner J.A., Nishimi K.M., Koenen K.C., Roberts A.L., Kubzansky L.D. (2020). Posttraumatic Stress Disorder and Inflammation: Untangling Issues of Bidirectionality. Biol. Psychiatry.

[9] American Psychiatric Association (1980). Diagnostic and Statistical Manual of Mental Disorders.

[10] American Psychiatric Association (1987). Diagnostic and Statistical Manual of Mental Disorders.

[11] American Psychiatry Association (2013). Diagnostic and Statistical Manual of Mental Health Disorders.

[12] Blais R.K., Tirone V., Orlowska D., Lofgreen A., Klassen B., Held P., Stevens N., Zalta A.K. (2021). Self-reported PTSD symptoms and social support in U.S. military service members and veterans: A meta-analysis. Eur. J. Psychotraumatol..

[13] Vasterling J.J., Proctor S.P., Friedman M.J., Hoge C.W., Heeren T., King L.A., King D.W. (2010). PTSD symptom increases in Iraq-deployed soldiers: Comparison with non-deployed soldiers and associations with baseline symptoms, deployment experiences, and postdeployment stress. J. Trauma. Stress.

[14] Xue C., Ge Y., Tang B., Liu Y., Kang P., Wang M., Zhang L.A. (2013). Meta-analysis of risk factors for combat-related PTSD among military personnel and veterans. PLoS ONE.

[15] Hoge C.W., Riviere L.A., Wilk J.E., Herrell R.K., Weathers F.W. (2014). The prevalence of post-traumatic stress disorder (PTSD) in US combat soldiers: A head-to-head comparison of DSM-5 versus DSM-IV-TR symptom criteria with the PTSD checklist. Lancet Psychiatry.

[16] Karam E.G., Friedman M.J., Hill E.D., Kessler R.C., McLaughlin K.A., Petukhova M., Sampson L., Shahly V., Angermeyer M.C., Bromet E.J. (2014). Cumulative traumas and risk thresholds: 12-month PTSD in the World Mental Health (WMH) surveys. Depress. Anxiety.

[17] Pietrzak R.H., Goldstein R.B., Southwick S.M., Grant B.F. (2011). Prevalence and Axis I comorbidity of full and partial posttraumatic stress disorder in the United States: Results from Wave 2 of the National Epidemiologic Survey on Alcohol and Related Conditions. J. Anxiety Disord..

[18] Sarapultsev A., Sarapultsev P., Dremencov E., Komelkova M., Tseilikman O., Tseilikman V. (2020). Low glucocorticoids in stress-related disorders: The role of inflammation. Stress.

[19] Sherin J.E., Nemeroff C.B. (2011). Post-traumatic stress disorder: The neurobiological impact of psychological trauma. Dialogues Clin. Neurosci..

[20] Baker D.G., Ekhator N.N., Kasckow J.W., Dashevsky B., Horn P.S., Bednarik L., Geracioti T.D. (2005). Higher levels of basal serial CSF cortisol in combat veterans with posttraumatic stress disorder. Am. J. Psychiatry.

[21] Sautter F.J., Bissette G., Wiley J., Manguno-Mire G., Schoenbachler B., Myers L., Johnson J.E., Cerbone A., Malaspina D. (2003). Corticotropin-releasing factor in posttraumatic stress disorder (PTSD) with secondary psychotic symptoms, nonpsychotic PTSD, and healthy control subjects. Biol. Psychiatry.

[22] Gjerstad J.K., Lightman S.L., Spiga F. (2018). Role of Glucocorticoid Negative Feedback in the Regulation of HPA Axis Pulsatility. Stress.

[23] Gola H., Engler A., Morath J., Adenauer H., Elbert T., Kolassa I.T., Engler H. (2014). Reduced peripheral expression of the glucocorticoid receptor alpha isoform in individuals with posttraumatic stress disorder: A cumulative effect of trauma burden. PLoS ONE.

[24] Gillespie C.F., Phifer J., Bradley B., Ressler K.J. (2009). Risk and resilience: Genetic and environmental influences on development of the stress response. Depress. Anxiety.

[25] Polanczyk G., Caspi A., Williams B., Price T.S., Danese A., Sugden K., Uher F., Poulton R., Moffitt T.E. (2009). Protective effect of CRHR1 gene variants on the development of adult depression following childhood maltreatment. Arch. Gen. Psychiatry.

[26] Amstadter A.B., Nugent N.R., Yang B.Z., Miller A., Siburian R., Moorjani P., Haddad S., Basu A., Fagerness J., Saxe G. (2011). Corticotrophin-releasing hormone type 1 receptor gene (CRHR1) variants predict posttraumatic stress disorder onset and course in pediatric injury patients. Dis. Markers.

[27] Yehuda R. (2006). Advances in understanding neuroendocrine alterations in PTSD and their therapeutic implication. Ann. N. Y. Acad. Sci..

[28] Yehuda R. (2009). Status of Glucocorticoid Alterations in Post-traumatic Stress Disorder. Ann. N. Y. Acad. Sci..

[29] Labonté B., Azoulay N., Yerko V., Turecki G., Brunet A. (2014). Epigenetic modulation of glucocorticoid receptors in posttraumatic stress disorder. Transl. Psychiatry.

[30] Martins C.S., Elias D., Colli L.M., Couri C.E., Souza M.C., Moreira A.C., Foss M.C., Elias L.L., de Castro M. (2017). HPA axis dysregulation, NR3C1 polymorphisms and glucocorticoid receptor isoforms imbalance in metabolic syndrome. Diabetes Metab. Res. Rev..

[31] Vukojevic V., Kolassa I.T., Fastenrath M., Gschwind L., Spalek K., Milnik A., Heck A., Vogler C., Wilker S., Demougin P. (2014). Epigenetic modification of the glucocorticoid receptor gene is linked to traumatic memory and post-traumatic stress disorder risk in genocide survivors. J. Neurosci..

[32] Yehuda R., Flory J.D., Bierer L.M., Henn-Haase C., Lehrner A., Desarnaud F., Makotkine I., Daskalakis N.P., Marmar C.R., Meaney M.J. (2015). Lower methylation of glucocorticoid receptor gene promoter 1F in periph eral blood of veterans with posttraumatic stress disorder. Biol. Psychiatry.

[33] Rohleder N., Wolf J.M., Wolf O.T. (2010). Glucocorticoid sensitivity of cognitive and inflammatory processes in depression and posttraumatic stress disorder. Neurosci. Biobehav. Rev..

[34] De Kloet C.S., Vermetten E., Bikker A., Meulman E., Geuze E., Kavelaars A., Westenberg H.G., Heijnen C.J. (2007). Leukocyte glucocorticoid receptor expression and immunoregulation in veterans with and without post-traumatic stress disorder. Mol. Psychiatry.

[35] Mahan A.L., Ressler K.J. (2012). Fear conditioning, synaptic plasticity and the amygdala: Implications for posttraumatic stress disorder. Trends Neurosci..

[36] Resnik J., Paz R. (2015). Fear generalization in the primate amygdala. Nat. Neurosci..

[37] Etkin A., Wager T.D. (2007). Functional neuroimaging of anxiety: A meta-analysis of emotional processing in PTSD, social anxiety disorder, and specific phobia. Am. J. Psychiatry.

[38] Linnman C., Zeffiro T.A., Pitman R.K., Milad M.R. (2011). An fMRI study of unconditioned responses in post-traumatic stress disorder. Biol. Mood Anxiety Disord..

[39] Smith N.B., Doran J.M., Sippel L.M., Harpaz-Rotem I. (2017). Fear extinction and memory reconsolidation as critical components in behavioral treatment for posttraumatic stress disorder and potential augmentation of these processes. Neurosci. Lett..

[40] Kuo J.R., Kaloupek D.G., Woodward S.H. (2012). Amygdala volume in combat-exposed veterans with and without posttraumatic stress disorder: A cross-sectional study. Arch. Gen. Psychiatry.

[41] Morey R.A., Gold A.L., Labar K.S., Beall S.K., Brown V.M., Haswell C.C., Nasser J.D., Wagner H.R., McCarthy G. (2012). Mid-Atlantic MIRECC Workgroup Amygdala volume changes with posttraumatic stress disorder in a large case-controlled veteran group. Arch. Gen. Psychiatry.

[42] Muscatell K.A., Dedovic K., Slavich G.M., Jarcho M.R., Breen E.C., Bower J.E., Eisenberger N.I. (2015). Greater amygdala activity and dorsomedial prefrontal-amygdala coupling are associated with enhanced inflammatory responses to stress. Brain Behav. Immun..

[43] Ehrlich D.E., Josselyn S.A. (2016). Plasticity-related genes in brain development and amygdala-dependent learning. Genes Brain Behav..

[44] McEwen B.S., Eiland L., Hunter R.G., Miller M.M. (2012). Stress and anxiety: Structural plasticity and epigenetic regulation as a consequence of stress. Neuropharmacology.

[45] Stevens J.S., Jovanovic T., Fani N., Ely T.D., Glover E.M., Bradley B., Ressler K. (2013). Disrupted amygdala-prefrontal functional connectivity in civilian women with posttraumatic stress disorder. J. Psychiatr. Res..

[46] Shin L.M., Orr S.P., Carson M.A., Rauch S.L., Macklin M.L., Lasko N.B., Peters P.M., Metzger L.J., Dougherty D.D., Cannistraro P.A. (2004). Regional cerebral blood flow in the amygdala and medial prefrontal cortex during traumatic imagery in male and female Vietnam veterans with PTSD. Arch. Gen. Psychiatry.

[47] Berlim M.T., Van den Eynde F. (2014). Repetitive transcranial magnetic stimulation over the dorsolateral prefrontal cortex for treating posttraumatic stress disorder: An exploratory meta-analysis of randomized, double-blind and sham-controlled trials. Can. J. Psychiatry.

[48] Woodward S.H., Kaloupek D.G., Streeter C.C., Martinez C., Schaer M., Eliez S. (2006). Decreased anterior cingulate volume in combat-related PTSD. Biol. Psychiatry.

[49] Koenigs M., Grafman J. (2009). Posttraumatic stress disorder: The role of medial prefrontal cortex and amygdala. Neuroscientist.

[50] Koch S.B., Van Zuiden M., Nawijn L., Frijling J.L., Veltman D.J., Olff M. (2017). Decreased uncinate fasciculus tract integrity in male and female patients with PTSD: A diffusion tensor imaging study. J. Psychiatry Neurosci..

[51] Sah P., Faber E.S., Lopez De Armentia M., Power J. (2003). The amygdaloid complex: Anatomy and physiology. Physiol. Rev..

[52] Milad M.R., Pitman R.K., Ellis C.B., Gold A.L., Shin L.M., Lasko N.B., Zeidan M.A., Handwerger K., Orr S.P., Rauch S.L. (2009). Neurobiological Basis of Failure to Recall Extinction Memory in Posttraumatic Stress Disorder. Biol. Psychiatry.

[53] Diener S.J., Nees F., Wessa M., Wirtz G., Frommberger U., Penga T., Ruttorf M., Ruf M., Schmahl C., Flor H. (2016). Reduced amygdala responsivity during conditioning to trauma-related stimuli in posttraumatic stress disorder: Conditioning to trauma-related stimuli in PTSD. Psychophysiology.

[54] Del Casale A., Ferracuti S., Barbetti A.S., Bargagna P., Zega P., Iannuccelli A., Caggese F., Zoppi T., De Luca G.P., Parmigiani G. (2022). Grey Matter Volume Reductions of the Left Hippocampus and Amygdala in PTSD: A Coordinate-Based Meta-Analysis of Magnetic Resonance Imaging Studies. Neuropsychobiology.

[55] Stevens J.S., Reddy R., Kim Y.J., van Rooij S.J.H., Ely T.D., Hamann S., Ressler K.J., Jovanovica T. (2018). Episodic memory after trauma exposure: Medial temporal lobe function is positively related to re-experiencing and inversely related to negative affect symptoms. NeuroImage.

[56] Lanius R.A., Williamson P.C., Densmore M., Boksman K., Gupta M.A., Neufeld R.W., Gati J.S., Menon R.S. (2011). Neural correlates of traumatic memories in posttraumatic stress disorder: A functional MRI investigation. Am. J. Psychiatry.

[57] Rogers M.A., Yamasue H., Abe O., Yamada H., Ohtani T., Iwanami A., Aoki S., Kato N., Kasai K. (2009). Smaller amygdala volume and reduced anterior cingulate gray matter density associated with history of post-traumatic stress disorder. Psychiatry Res.-Neuroimaging.

[58] Wignall E.L., Dickson J.M., Vaughan P., Farrow T.F.D., Wilkinson I.D., Hunter M.D., Woodruff P.W.R. (2004). Smaller hippocampal volume in patients with recent-onset posttraumatic stress disorder. Biol. Psychiatry.

[59] Morey R.A., Clarke E.K., Haswell C.C., Phillips R.D., Clausen A.N., Mufford M.S., Saygin Z. (2020). Amygdala Nuclei Volume and Shape in Military Veterans with Posttraumatic Stress Disorder. VA Mid-Atlantic MIRECC Workgroup, Wagner HR, LaBar KS. Biol. Psychiatry Cogn. Neurosci. Neuroimaging.

[60] Bird C.M., Burgess N. (2008). The hippocampus and memory: Insights from spatial processing. Nat. Rev. Neurosci..

[61] Quidé Y., Andersson F., Dufour-Rainfray D., Descriaud C., Brizard B., Gissot V., Cléry H., Carrey Le Bas M.-P., Osterreicher S., Ogielska S. (2018). Smaller hippocampal volume following sexual assault in women is associated with post-traumatic stress disorder. Acta Psychiatr. Scand..

[62] Shin L.M., Shin P.S., Heckers S., Krangel T.S., Macklin M.L., Orr S.P., Lasko N., Segal E., Makris N., Richert K. (2004). Hippocampal function in posttraumatic stress disorder. Hippocampus.

[63] Akiki T.J., Averill C.L., Wrocklage K.M., Schweinsburg B., Scott J.C., Martini B., Averill L.A., Southwick S.M., Krystal J.H., Abdallah C.G. (2017). The association of PTSD symptom severity with localized hippocampus and amygdala abnormalities. Chronic Stress.

[64] Jacob Y., Morris L.S., Verma G., Rutter S.B., Balchandani P., Murrough J.W. (2022). Altered hippocampus and amygdala subregion connectome hierarchy in major depressive disorder. Transl. Psychiatry.

[65] Clelland C.D., Choi M., Romberg C., Clemenson G.D., Fragniere A., Tyers P., Jessberger S., Saksida L.M., Barker R.A., Gage F.G. (2009). A functional role for adult hippocampal neurogenesis in spatial pattern separation. Science.

[66] Hayes J.P., Hayes S., Miller D.R., Lafleche G., Logue M.W., Verfaellie M. (2017). Automated measurement of hippocampal subfields in PTSD: Evidence for smaller dentate gyrus volume. J. Psychiatr. Res..

[67] Cohen H., Kozlovsky N., Matar M.A., Zohar J., Kaplan Z. (2014). Distinctive hippocampal and amygdalar cytoarchitectural changes underlie specific patterns of behavioral disruption following stress exposure in an animal model of PTSD. Eur. Neuropsychopharmacol..

[68] Boldrini M., Santiago A.N., Hen R., Dwork A.J., Rosoklija G.B., Tamir H., Arango V., John M.J. (2013). Hippocampal granule neuron number and dentate gyrus volume in antidepressant-treated and untreated major depression. Neuropsychopharmacology.

[69] Renault P.F., Hoofnagle J.H., Park Y., Mullen K.D., Peters M., Jones B., Rustgi V., Jones A. (1987). Psychiatric complications of long-term interferon alfa therapy. Arch. Intern. Med..

[70] Dowlati Y., Herrmann N., Swardfager W., Liu H., Sham L., Reim E.K., Lanctot K.L. (2010). A meta-analysis of cytokines in major depression. Biol. Psychiatry.

[71] Dinarello C.A. (2000). Proinflammatory cytokines. Chest.

[72] Lindqvist D., Wolkowitz O.M., Mellon S., Yehuda R., Flory J.D., Henn-Haase C., Bierer L.M., Abu-Amara D., Coy M., Neylan T.C. (2014). Proinflammatory milieu in combat-related PTSD is independent of depression and early life stress. Brain Behav. Immun..

[73] Lindqvist D., Dhabhar F.S., Mellon S.H., Yehuda R., Grenon S.M., Flory J.D., Bierer L.M., Abu-Amara D., Coy M., Makotkine I. (2017). Increased pro-inflammatory milieu in combat related PTSD: A new cohort replication study. Brain Behav. Immun..

[74] Miller K., Driscoll D., Smith L.M., Ramaswamy S. (2017). The role of inflammation in late-life post-traumatic stress disorder. Mil. Med..

[75] Imai R., Hori H., Itoh M., Lin M., Niwa M., Ino K., Ogawa S., Ishida M., Sekiguchi A., Matsui M. (2018). Inflammatory markers and their possible effects on cognitive function in women with posttraumatic stress disorder. J. Psychiatr. Res..

[76] De Oliveira J.F., Wiener C.D., Jansen K., Portela L.V., Lara D.R., Souza L.D.D.M., da Silva R.A., Moreira F.P., Oses J.P. (2018). Serum levels of interleukins IL-6 and IL-10 in individuals with posttraumatic stress disorder in a population-based sample. Psychiatry Res..

[77] Bruenig D., Mehta D., Morris C.P., Harvey W., Lawford B., Young R.M., Voisey J. (2017). Genetic and serum biomarker evidence for a relationship between TNFα and PTSD in Vietnam War combat veterans. Compr. Psychiatry.

[78] Passos I.C., Vasconcelos-Moreno M.P., Costa L.G., Kunz M., Briezke E., Quewvedo J., Salum G., Magalhaes P.V., Kapczinski F., Kauer-SantAnna M. (2015). Inflammatory markers in post-traumatic stress disorder: A systematic review, meta-analysis, and meta-regression. Lancet Psychiatry.

[79] Logue M.W., Baldwin C., Guffanti G., Melista E., Wolf E.J., Reardon A.F., Uddin M., Wildman D., Galea S., Koenen K.C. (2013). A genome-wide association study of post-traumatic stress disorder identifies the retinoid-related orphan receptor alpha (RORA) gene as a significant risk locus. Mol. Psychiatry.

[80] Friend S.F., Nachnani R., Powell S.B., Risbrough V.B. (2020). C-Reactive Protein: Marker of risk for post-traumatic stress disorder and its potential for a mechanistic role in trauma response and recovery. Eur. J. Neurosci..

[81] Rosen R.L., Levy-Carrick N., Reibman J., Xu N., Shao Y., Liu M., Ferri L., Kazeros A., Caplan-Shaw C.E., Pradhan D.R. (2017). Elevated C-reactive protein and posttraumatic stress pathology among survivors of the 9/11 World Trade Center attacks. J. Psychiatr. Res..

[82] Baumert J., Lukaschek K., Kruse J., Emeny R.T., Koenig W., von Kanel R., Ladwig K.H. (2013). investigators K. No evidence for an association of posttraumatic stress disorder with circulating levels of CRP and IL-18 in a population-based study. Cytokine.

[83] Maples-Keller J.L., Yasinski C., Stojek M., Ravi M., Watkins L.E., Patton S.C., Rothbaum A.O., Unongo M., Dunlop B.W., Rauch S.A.M. (2022). The relations between C-reactive protein and trauma exposure, PTSD and depression symptoms, and PTSD psychotherapy treatment response in treatment seeking veterans and service members. Brain Behav. Immun..

[84] McD Young R., Lawford B., Mellor R., Morris C.P., Voisey J. (2021). PTSD Initiative. Investigation of C-reactive protein and AIM2 methylation as a marker for PTSD in Australian Vietnam veterans. Gene.

[85] Michopoulos V., Rothbaum A.O., Jovanovic T., Almli L.M., Bradley B., Rothbaum B.O., Gillespie C.F., Ressler K.J. (2015). Association of CRP genetic variation and CRP level with elevated PTSD symptoms and physiological responses in a civilian population with high levels of trauma. Am. J. Psychiatry.

[86] Miller M.W., Maniates H., Wolf E.J., Logue M.W., Schichman S.A., Stone A., Milberg W., McGlinchey R. (2018). CRP polymorphisms and DNA methylation of the AIM2 gene influence associations between trauma exposure, PTSD, and C-reactive protein. Brain Behav. Immun..

[87] Speer K., Upton D., Semple S., McKune A. (2018). Systemic low-grade inflammation in post-traumatic stress disorder: A systematic review. J. Inflamm. Res..

[88] Daskalakis N.P., Cohen H., Nievergelt C.M., Baker D.G., Buxbaum J.D., Russo S.J., Yehuda R. (2016). New translational perspectives for blood-based biomarkers of PTSD: From glucocorticoid to immune mediators of stress susceptibility. Exp. Neurol..

[89] Coutinho A.E., Chapman K.E. (2011). The anti-inflammatory and immunosuppressive effects of glucocorticoids, recent developments and mechanistic insights. Mol. Cell. Endocrinol..

[90] Hendrickson R.C., Raskind M.A., Millard S.P., Sikkema C., Terry G.E., Pagulayan K.F., Li G., Peskind E.R. (2018). Evidence for altered brain reactivity to norepinephrine in Veterans with a history of traumatic stress. Neurobiol. Stress.

[91] Fleshner M., Frank M., Maier S.F. (2017). Danger signals and inflammasomes: Stress- evoked sterile inflammation mood disorders. Neuropsychopharmacology.

[92] Franklin T.C., Xu C., Duman R.S. (2018). Depression and sterile inflammation: Essential role of danger associated molecular patterns. Brain Behav. Immun..

[93] Trapero I., Cauli O. (2014). Interleukin 6 and cognitive dysfunction. Metab. Brain Dis..

[94] Mellon S.H., Gautman A., Hammamieh R., Jett M., Wolkowitz O.M. (2018). Metabolism, motabolomics, and inflammation in posttraumatic stress disorder. Biol. Psychiatry.

[95] Sen R., Baltimore D. (1986). Inducibility of kappa immunoglobulin enhancer-binding protein NF-kappa B by a posttranslational mechanism. Cell.

[96] Kaltschmidt B., Widera D., Kaltschmidt C. (2005). Signaling via NF-kappaB in the nervous system. Biochim. Biophys. Acta.

[97] Mattson M.P., Meffert M.K. (2006). Roles for NF-kappa B in nerve cell survival, plasticity, and disease. Cell Death Differ..

[98] Meffert M.K., Baltimore D. (2005). Physiological functions for brain NF-kappaB. Trends Neurosci..

[99] Dresselhaus E.C., Meffert M.K. (2019). Cellular Specificity of NF-κB Function in the Nervous System. Front. Immunol..

[100] Baeuerle P.A., Baltimore D. (1988). Kappa B: A specific inhibitor of the NF-kappa B transcription factor. Science.

[101] Ben-Neriah Y. (2002). Regulatory functions of ubiquitination in the immune system. Nat. Immunol..

[102] Ghosh S., May M.J., Kopp E.B. (1998). NF-κB and Rel proteins: Evolutionary conserved mediators of immune responses. Annu. Rev. Immunol..

[103] Caamano J., Hunter C.A. (2002). NF-κB family of transcription factors: Central regulators of innate and adaptive immune functions. Clin. Microbiol. Rev..

[104] Baeuerle P., Baltimore D. (1996). NF-B: Ten years after. Cell Death Differ..

[105] De Bosscher K., Haegeman G. (2009). Minireview: Latest perspectives on antiinflammatory actions of glucocorticoids. Mol. Endocrinol..

[106] Mattson M.P., Culmsee C., Yu Z., Camandola S. (2000). Roles of nuclear factor κB in neuronal survival and plasticity. J. Neurochem..

[107] Mattson M.P., Goodman Y., Luo H., Fu W., Furukawa K. (1997). Activation of NF-κB protects hippocampal neurons against oxidative stress-induced apoptosis: Evidence for induction of manganese superoxide dismutase and suppression of peroxynitrite production and protein tyrosine nitration. J. Neurosci. Res..

[108] Meberg P.J., Kinney W.R., Valcourt E.G., Routtenberg A. (1996). Gene expression of the transcription factor NF-κB in hippocampus: Regulation by synaptic activity. Brain Res. Mol. Brain Res..

[109] Howell J.A., Bidwell G.L. (2020). Targeting the NF-kappaB pathway for therapy of ischemic stroke. Ther. Deliv..

[110] Ridder D.A., Schwaninger M. (2009). NF-kappaB signaling in cerebral ischemia. Neuroscience.

[111] Singh S., Singh T.G. (2020). Role of Nuclear Factor Kappa B (NF-kappaB) Signalling in Neurodegenerative Diseases: A Mechanistic Approach. Curr. Neuropharmacol..

[112] Mettang M., Reichel S.N., Lattke M., Palmer A., Abaei A., Rasche V., Huber-Lang M., Baumann B. (2018). Wirth T IKK2/NF-kappaB signaling protects neurons after traumatic brain injury. FASEB J..

[113] Schneider A., Martin-Villalba A., Weih F., Vogel J., Wirth T., Schwaninger M. (1999). NF-kappaB is activated and promotes cell death in focal cerebral ischemia. Nat. Med..

[114] Miller J.A., Kirkley K.A., Padmanabhan R., Liang L.P., Raol Y.H., Patel M., Bialecki R.A., Tjalkens R.B. (2014). Repeated exposure to low doses of kainic acid activates nuclear factor kappa B (NF-kappaB) prior to seizure in transgenic NF-kappaB/EGFP reporter mice. Neurotoxicology.

[115] Bhakar A.L., Tannis L.L., Zeindler C., Russo M.P., Jobin C., Park D.S., MacPherson S., Barker P.A. (2002). Constitutive nuclear factor-kappa B activity is required for central neuron survival. J. Neurosci..

[116] Fridmacher V., Kaltschmidt B., Goudeau B., Ndiaye D., Rossi F.M., Pfeiffer J., Kaltschmidt C., Israël A., Mémet S. (2003). Forebrain-specific neuronal inhibition of nuclear factor-kappaB activity leads to loss of neuroprotection. J. Neurosci..

[117] Kassed C.A., Herkenham M. (2004). NF-kappaB p50-deficient mice show reduced anxiety-like behaviors in tests of exploratory drive and anxiety. Behav. Brain Res..

[118] Kook J.W., Russo S.J., Ferguson D., Nestler E.J., Duman R.S. (2010). Nuclear factor-kappaB is a critical mediator of stress-impaired neurogenesis and depressive behavior. Proc. Natl. Acad. Sci. USA.

[119] Cohen H., Kozlovsky N., Matar M.A., Zohar J., Kaplan Z. (2011). The characteristic long-term upregulation of hippocampal NF-kappaB complex in PTSD-like behavioral stress response is normalized by high-dose corticosterone and pyrrolidine dithiocarbamate administered immediately after exposure. Neuropsychopharmacology.

[120] Pace T.W., Mletzko T.C., Alagbe O., Musselman D.L., Nemeroff C.B., Miller A.H., Heim C.M. (2006). Increased stress-induced inflammatory responses in male patients with major depression and increased early life stress. Am. J. Psychiatry.

[121] O’Donovan A., Sun B., Cole S., Rempel H., Lenoci M.L., Pulliam T. (2011). Neylan Transcriptional control of monocyte gene expression in post-traumatic stress disorder. Dis. Markers.

[122] Galic M.A., Riazi K., Pittman Q.J. (2012). Cytokines and brain excitability. Front. Neuroendocrinol..

[123] Tan K.S., Nackley A.G., Satterfield K., Maixner W., Diatchenko L., Flood P.M. (2007). Beta2 adrenergic receptor activation stimulates pro-inflammatory cytokine production in macrophages via PKA- and NF-kappaB-independent mechanisms. Cell. Signal..

[124] Hollenberg S.M., Weinberger C., Ong E.S., Cerelli G., Oro A., Lebo R., Thompson E.B., Rosenfeld M.G., Evans R.M. (1985). Primary structure and expression of a functional human glucocorticoid receptor cDNA. Nature.

[125] Silverman M.N., Sternberg E.M. (2012). Glucocorticoid regulation of inflammation and its functional correlates: From HPA axis to glucocorticoid receptor dysfunction. Ann. N. Y. Acad Sci..

[126] Harris A.P., Holmes M.C., de Kloet E.R., Chapman K.E., Seckl J.R. (2013). Mineralocorticoid and glucocorticoid receptor balance in control of HPA axis and behavior. Psychoneuroendocrinology.

[127] Hollenberg S.M., Evans R.M. (1998). Multiple and cooperative trans-activation domains of the human glucocorticoid receptor. Cell.

[128] Hong H., Kohli K., Garabedian M.J., Stallcup M.R. (1997). GRIP1, a transcriptional coactivator for the AF-2 transactivation domain of steroid, thyroid, retinoid, and vitamin D receptors. Mol. Cell. Biol..

[129] Ray A., Prefontaine K.E. (1994). Physical association and functional antagonism between the p65 subunit of transcription factor NF-κB and the glucocorticoid receptor. Proc. Natl. Acad. Sci. USA.

[130] Caldenhoven E., Liden J., Wissink S., van de Stolpe A., Raaijmakers J., Koenderman L., Okret S., Gustafsson J.A., van der Saag P.T. (1995). Negative cross-talk between RelA and the glucocorticoid receptor: A possible mechanism for the antiinflammatory action of glucocorticoids. Mol. Endocrinol..

[131] Scheinman R.I., Gualberto A., Jewell C.M., Cidlowski J.A., Baldwin A.S. (1995). Characterization of mechanisms involved in transrepression of NF-kB by activated glucocorticoid receptors. Mol. Cell. Biol..

[132] De Bosscher K., Schmitz M.L., Vanden Berghe W., Plaisance S., Fiers W., Haegeman G. (1997). Glucocorticoid-mediated repression of nuclear factor-kappaB-dependent transcription involves direct interference with transactivation. Proc. Natl. Acad. Sci. USA.

[133] Boersma M.C., Dresselhaus E.C., De Biase L.M., Mihalas A.B., Bergles D.E., Meffert M.K. (2011). A requirement for nuclear factor-kappaB in developmental and plasticity-associated synaptogenesis. J. Neurosci..

[134] Kim E.J., Pellman B., Kim J.J. (2015). Stress effects on the hippocampus: A critical review. Learn Mem..

[135] Krystal J.H., Abdallah C.G., Averill L.A., Kelmendi B., Harpaz-Rotem I., Sanacora G., Southwick S.M., Duman R.S. (2017). Synaptic Loss and the Pathophysiology of PTSD: Implications for Ketamine as a Prototype Novel Therapeutic. Curr. Psychiatry Rep..

[136] Golia M.T., Poggini S., Alboni S., Garofalo S., Ciano Albanese N., Viglione A., Ajmone-Cat M.A., St-Pierre A., Brunello N., Limatola C. (2019). Interplay between inflammation and neural plasticity: Both immune activation and suppression impair LTP and BDNF expression. Brain Behav. Immun..

[137] Krystal J.H., Sanacora G., Duman R.S. (2013). Rapid-acting glutamatergic antidepressants: The path to ketamine and beyond. Biol. Psychiatry.

[138] Carrillo-de Sauvage M.A., Maatouk L., Arnoux I., Pasco M., Sanz Diez A., Delahaye M., Herrero M.T., Newman T.A., Calvo C.F., Audinat E. (2013). Potent and multiple regulatory actions of microglial glucocorticoid receptors during CNS inflammation. Cell Death Differ..

[139] Meffert M.K., Chang J.M., Wiltgen B.J., Fanselow M.S., Baltimore D. (2003). NF-kappa B functions in synaptic signaling and behavior. Nat. Neurosci..

[140] Miller G.E., Cohen S., Ritchey A.K. (2002). Chronic psychological stress and the regulation of proinflammatory cytokines: A glucocorticoid-resistance model. Health Psychol..

[141] Miller G.E., Murphy M.L., Cashman R., Ma R., Ma J., Arevalo J.M., Kobor M.S., Cole S.W. (2014). Greater inflammatory activity and blunted glucocorticoid signaling in monocytes of chronically stressed caregivers. Brain Behav. Immun..

[142] Snow W.M., Stoesz B.M., Kelly D.M., Albensi B.C. (2014). Roles for NF-κB and gene targets of NF-κB in synaptic plasticity, memory, and navigation. Mol. Neurobiol..

[143] Freudenthal R., Romano A., Routtenberg A. (2004). Transcription factor NF-kappaB activation after in vivo perforant path LTP in mouse hippocampus. Hippocampus.

[144] Albensi B.C., Mattson M.P. (2000). Evidence for the involvement of TNF and NF-kappaB in hippocampal synaptic plasticity. Synapse.

[145] Oikawa K., Odero G.L., Platt E., Neuendorff M., Hatherell A., Bernstein M.J., Albensi B.C. (2012). NF-κB p50 subunit knockout impairs late LTP and alters long term memory in the mouse hippocampus. BMC Neurosci..

[146] Munhoz C.D., Lepsch L.B., Kawamoto E.M., Malta M.B., Lima L.d.S., Avellar M.C., Sapolsky R.M., Scavone C. (2006). Chronic unpredictable stress exacerbates lipopolysaccharide-induced activation of nuclear factor-kappaB in the frontal cortex and hippocampus via glucocorticoid secretion. J. Neurosci..

[147] Boccia M., Freudenthal R., Blake M., de la Fuente V., Acosta G., Baratti C., Romano A. (2007). Activation of hippocampal nuclear factor-kappa B by retrieval is required for memory reconsolidation. J. Neurosci..

[148] Freudenthal R., Boccia M.M., Acosta G.B., Blake M.G., Merlo E., Baratti C.M., Romano A. (2005). NF-kappaB transcription factor is required for inhibitory avoidance long-term memory in mice. Eur. J. Neurosci..

[149] Yang J., Yu J., Jia X., Zhu W., Zhao L., Li S., Xu C., Yang C., Wu P., Lu L. (2011). Inhibition of nuclear factor-kappaB impairs reconsolidation of morphine reward memory in rats. Behav. Brain Res..

[150] Ahn H.J., Hernandez C.M., Levenson J.M., Lubin F.D., Liou H.C., Sweatt J.D. (2008). c-Rel, an NF-kappaB family transcription factor, is required for hippocampal long-term synaptic plasticity and memory formation. Learn Mem..

[151] Kaltschmidt B., Ndiaye D., Korte M., Pothion S., Arbibe L., Prüllage M., Pfeiffer J., Lindecke A., Staiger V., Israël A. (2006). NF-kappaB regulates spatial memory formation and synaptic plasticity through protein kinase A/CREB signaling. Mol. Cell. Biol..

[152] Si J., Yang J., Xue L., Yang C., Luo Y., Shi H., Lu L. (2012). Activation of NF-kB in Basolateral Amygdala Is Required for Memory Reconsolidation in Auditory Fear Conditioning. PLoS ONE.

[153] Koss K., Churchward M.A., Tsui C., Todd K.G. (2019). In vitro priming and hyper-activation of brain microglia: An assessment of phenotypes. Mol. Neurobiol..

[154] Zhang Z., Song Z., Shen F., Xie P., Wang J., Zhu A.S., Zhu G. (2021). Ginsenoside Rg1 prevents PTSD-like behaviors in mice through promoting synaptic proteins, reducing Kir4.1 and TNF-α in the hippocampus. Mol. Neurobiol..

[155] Li S., Liao Y., Dong Y., Li X., Li J., Cheng Y., Cheng J., Yuan Z. (2021). Microglial deletion and inhibition alleviate behavior of post-traumatic stress disorder in mice. J. Neuroinflamm..

